# Naturally occurring basal core promoter A1762T/G1764A dual mutations increase the risk of HBV-related hepatocellular carcinoma: a meta-analysis

**DOI:** 10.18632/oncotarget.7123

**Published:** 2016-02-02

**Authors:** Zongguo Yang, Liping Zhuang, Yunfei Lu, Qingnian Xu, Bozong Tang, Xiaorong Chen

**Affiliations:** ^1^ Shanghai Public Health Clinical Center, Fudan University, Shanghai, China; ^2^ Fudan University Shanghai Cancer Center, Shanghai, China; ^3^ Shanghai Medical College, Fudan University, Shanghai, China

**Keywords:** hepatitis B virus X protein, hepatocellular carcinoma, basal core promoter, mutation, A1762T/G1764A

## Abstract

Basal core promoter (BCP) A1762T/G1764A dual mutations in hepatocarcinogenesis remain controversial. Published studies up to June 1, 2015 investigating the frequency of A1762T/G1764A dual mutations from chronic hepatitis B virus (HBV) infection, including hepatocellular carcinoma (HCC), were systematically identified. A total of 10,240 patients with chronic HBV infection, including 3729 HCC cases, were included in 52 identified studies. HCC patients had a higher frequency of BCP A1762T/G1764A dual mutations compared with asymptomatic HBsAg carriers (ASC) and patients with chronic hepatitis B (CHB) and liver cirrhosis (LC) (OR = 5.59, *P* < 0.00001; OR = 2.87, *P* < 0.00001; OR = 1.55, *P* = 0.02, respectively). No statistically significant difference was observed in the frequency of A1762T/G1764A dual mutations in cirrhotic HCC versus non-cirrhotic HCC patients (OR = 2.06, *P* = 0.05). Chronic HBV-infected patients and HCC patients with genotype B had a significantly lower risk of A1762T/G1764A dual mutations compared with patients with genotype C (OR = 0.30, *P* < 0.0001 and OR = 0.34, *P* = 0.04, respectively). In HBV genotype C subjects, A1762T/G1764A dual mutations contributed to significantly higher risk for HCC developing compared with non-mutation ones (OR = 3.47, *P* < 0.00001). In conclusion, A1762T/G1764A dual mutations increase the risk of HBV-related hepatocellular carcinoma, particularly in an HBV genotype C population, even without progression to cirrhosis.

## INTRODUCTION

Viral genomic mutations may contribute to HCC development. HBV nonstructural X protein (HBx) is a key regulatory protein of the virus and is at the intersection of HBV infection, replication, pathogenesis, and possibly carcinogenesis. HBx has different consequences for hepatocyte physiology because HBV-infected cells are targeted by the immune system or as hepatocytes, in which HBx is expressed, and undergo transformation and progression to HCC. In addition, HBx can influence apoptotic and cell cycle regulatory pathways [1].

However, the roles that HBx mutations play in hepatocarcinogenesis remain controversial, particularly for the basal core promoter (BCP) A1762T/G1764A dual mutation. Several prospective studies have demonstrated that patients with an A1762T/G1764A dual mutation were more predisposed to HCC than those with the wild type and that HBV mutations, including A1762T/G1764A, are associated with an increased risk of HCC [2]. However, a recent study using global data found no significant difference in BCP mutations between HCC and non-HCC patients with HBV genotype C, and the differences between chronic hepatitis B (CHB) and liver cirrhosis (LC) and between LC and HCC were not significant, although the mutant ratio increased with disease progression. [3].

In this meta-analysis, we summarized the prevalence of A1762T/G1764A mutations from ASC, CHB, LC and HCC patients. We also analyzed the BCP mutation rates in chronic HBV infection, including HCC patients grouped by HBV genotype and HBeAg status, hoping that the results might provide useful insights into the risk of HCC occurrence.

## RESULTS

### Study and patient characteristics

**Figure 1:**
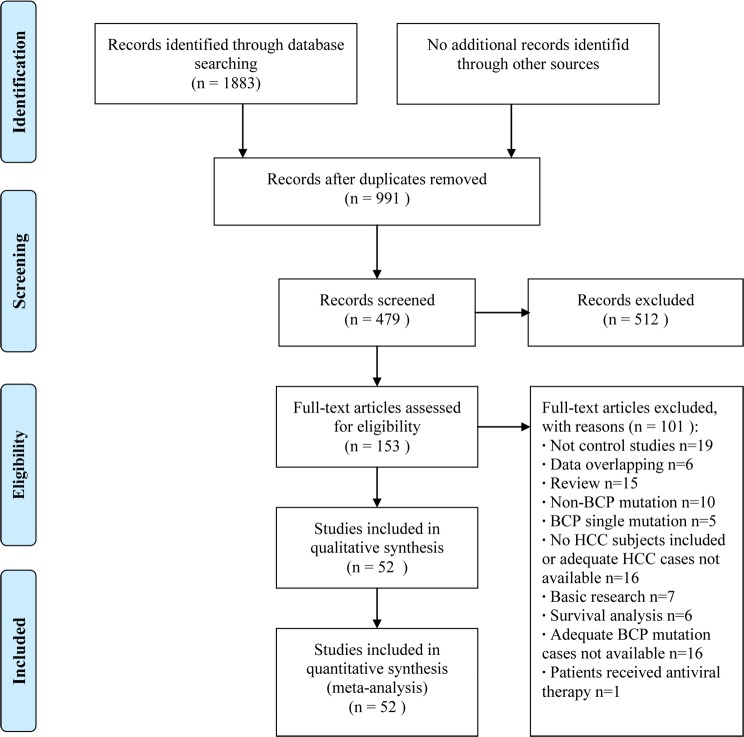
Flow program of study selection

A total of 1883 abstracts were reviewed. From these articles, 163 that were closely related to the current subject were retrieved. The study selection process is summarized in Figure 1. Finally, 52 case-control or cohort studies [4-55] were included in the meta-analysis. A total of 10,240 individuals with chronic HBV infection were included, 3729 of whom had HCC. The baseline characteristics and quality scores of the studies examined in this meta-analysis are listed in Table 2.

**Table 1:** Quality criteria for the included studies*

Quality parameter	Score		
	2	1	0
Study design	Cohort study or nested case-control study	Incidence case-control study. Prevalence case-control study	-
No. of case subjects	>100	50-100	<50
Source of population	Community-based or from two or more countries	≥2 hospitals	1 hospitals
Mutation detection method	DNA direct sequencing	Innogenetics line-probe assay	Single-strand conformation polymorphism; Mass spectrometer
Matching of case and control subjects			
Confounder group 1	Age and sex	Age or sex	None
Confounder group 2	HBeAg status and HBV genotype	HBeAg status or HBV genotype	None

* -, designs other than cohort or nested, incidence, or prevalence case-control not included in meta-analysis; HBeAg, hepatitis B e antigen; HBV, hepatitis B virus.

**Table 2:** Baseline characteristics of studies included in the meta-analysis

Study	Design	Country or area	No. of cases	No. of controls	Detection method	HBV genotype	Matching factors				Quality score
							Age	Sex	HbeAg status	HBV genotype	
Asim 2010	PCC	India	150	136	SSCP	A, D	-	-	-	-	≤4
Bahramali 2008	Cohort	Iran	7	55	Sequencing	D	-	-	-	-	≤4
Bai 2011	Cohort	China	152	136	Sequencing	B, C, others	-	-	-	-	7
Baptista 1999	PCC	South Africa	59	52	Sequencing	NA	+	-	-	-	5-7
Blackberg 2003	PCC	Sweden, others	16	19	Sequencing	A, B, C, D	-	+	+	+	≥8
Chen 2006	PCC	Taiwan	50	102	INNO-LiPA	B, C	+	-	-	-	5-7
Chen 2008	PCC	Taiwan	80	160	Sequencing	B, C, D	+	+	+	-	5-7
Chen 2009	PCC	Taiwan	222	300	Sequencing	B, C	+	+	-	-	5-7
Chen 2012	PCC	China	156	310	Sequencing	B, C	+	+	-	-	5-7
Cho 2011	PCC	Korea	69	125	Sequencing	C	-	-	-	-	≤4
Choi 2009	PCC	Korea	42	46	Sequencing	C	-	-	-	-	≤4
Chou 2008	NCC	Taiwan	154	316	Sequencing	A,B,C	+	-	-	-	≥8
Chu 2012	PCC	Taiwan	80	120	Sequencing	B, C	+	+	+	-	4-6
Constantinescu 2014	Cohort	Romania	94	390	Sequencing	A, D	-	-	-	-	5
Datta 2014	PCC	India	22	46	Sequencing	A, C, D	-	-	-	-	≤4
Elkady 2008	PCC	Mongolia	23	25	Sequencing	A, D	-	-	-	-	≤4
Fan 2011	PCC	China	34	38	Sequencing	B, C	+	+	+	-	5-7
Guo 2008	NCC	China	58	71	Sequencing	B, C	+	+	-	-	≥8
Ito 2006	ICC	Japan, United States, Hong Kong	40	80	Sequencing	C	+	+	+	+	≥8
Jang 2007	Cohort	Korea	6	23	Sequencing	C	-	-	+	+	≥8
Kao 2012	PCC	Taiwan	56	112	Sequencing	B, C	+	-	-	-	4-5
Kim 2008	PCC	Korea	60	124	Sequencing	C	-	-	-	+	5-7
Kim 2009	PCC	Korea	135	135	Sequencing	C	+	+	+	-	5-7
Kuang 2005	PCC	Thailand	34	68	Mass spectrometer	NA	+	+	-	-	≤4
Lee 2011	PCC	Korea	31	65	Sequencing	C	-	-	-	-	≤4
Li 2013	Cohort	China	102	105	Sequencing	C	-	-	-	-	6
Lin 2005	PCC	Taiwan	32	142	Sequencing	B, C, Others	-	-	+	-	≤4
Liu 2006	PCC	Taiwan	200	160	INNO-LiPA	B, C	-	-	-	-	≤4
Livingston 2007	ICC	United States	47	1129	INNO-LiPA	A, C, D, F	+	+	-	-	≤4
Lyu 2013	ICC	Korea	318	234	Sequencing	C	-	-	-	-	5
Malik 2012	PCC	India	118	294	Sequencing	A, D	-	-	-	-	5
Muroyama 2006	PCC	Japan	39	36	Sequencing	C	+	+	-	+	5-7
Panigrahi 2012	PCC	India	20	132	Sequencing	A, C, D	-	-	-	-	≤4
Park 2014	PCC	Korea	132	310	Sequencing	C	-	-	-	-	5
Qu 2014	ICC	China	152	131	Sequencing	B, C	+	-	-	-	5-7
Sakamoto 2006	PCC	Japan, Philippines	31	69	Sequencing	A, B, C	-	+	-	-	5-7
Shinkai 2007	PCC	Japan	80	80	Sequencing	C	+	+	+	+	≥8
Song 2005	PCC	Vietnam	48	74	Sequencing	NA	-	-	-	-	≤4
Tanaka 2006	PCC	Japan, Hong Kong	148	180	Sequencing	C	+	+	+	-	≥8
Tangkijvanich 2010	ICC	Thailand	60	60	Sequencing	B, C	+	+	+	+	6
Tong 2007	ICC	United States	101	67	Sequencing	A, B, C, D	-	-	-	-	5-7
Tong 2013	ICC	United States	173	240	Sequencing	A, B, C, D, E, F	+	-	-	-	5-7
Truong 2007	PCC	Japan, Vietnam	32	88	Sequencing	C	-	-	+	+	5-7
Utama 2009	PCC	Indonesia	48	123	Sequencing	B, C	-	-	-	-	5
Wang 2007	PCC	China	47	164	Sequencing	B, C	-	+	-	-	5-7
Xu 2010	PCC	China	60	120	Sequencing	A, B, C	+	+	-	-	4-6
Yin 2011	PCC	China	190	1269	Sequencing	B, C	+	+	-	-	≥8
Yuan 2007	PCC	China	34	207	Sequencing	B, C	-	+	-	+	5-7
Yuan 2009	ICC	China	49	97	Sequencing	NA	+	-	-	-	5-7
Zhang 2007	Cohort	China	32	32	Sequencing	NA	+	+	-	-	6-8
Zheng 2011	PCC	China	156	185	Sequencing	B, C, E	-	-	-	-	7
Zhu 2008	NCC	China	20	83	Sequencing	C	+	+	+	+	≥8

Abbreviations: NA, not available; PCC, prevalence case-control; NCC, nested case-control; ICC, incidence case-control; SSCP, Single-strand conformation polymorphism; INNO-LiPA, Innogenetics line-probe assay.

### Prevalence of A1762T/G1764A mutations from ASC, CHB, LC and HCC

Heterogeneity was significant among the included studies [4-38, 40-55] when comparing the prevalence of A1762T/G1764A dual mutations between HCC and non-HCC patients (*P* < 0.00001, I^2^ = 82%). Thus, a random-effects model was used. Our data revealed that more HCC patients had A1762T/G1764A dual mutations compared with non-HCC patients (66.5% vs. 39.8%, OR = 3.05, 95% CI = 2.35-3.95, *P* < 0.00001, Table 3 and Supplementary Figure S1).

**Table 3:** Frequency of BCP A1762T/G1764A dual mutations from chronic HBV-infected patients, including HCC

Comparisons	Studies	A1762T/G1764A dual mutation, n (%)	Heterogeneity				OR	95% CI	*P* value
			Chi^2^	df	P	I^2^ (%)			
HCC	[4-38, 40-55]	2480/3729 (66.5)	270.4	50	< 0.00001	82	3.05	2.35-3.95	< 0.00001
Non-HCC		2594/6511 (39.8)							
HCC	[5, 9, 12-14, 16, 18, 23, 25, 28, 30, 34, 36, 41, 46-48, 51, 55]	544/808 (67.3)	33.38	18	0.02	46	1.55	1.06-2.26	0.02
LC		441/708 (62.3)							
HCC	[5, 9, 12, 13, 20-23, 25, 28-30, 33, 34, 36, 41, 47, 48, 51, 53, 55]	927/1300 (71.3)	72.49	20	< 0.00001	72	2.87	1.96-4.20	< 0.00001
CHB		796/1566 (50.8)							
HCC	[5, 7-9, 16, 17, 22-25, 30, 31, 36, 41, 44, 51]	591/924 (64.0)	70.33	15	< 0.00001	79	5.59	3.17-9.83	< 0.00001
ASC		246/1068 (23.0)							
LC-HCC	[12, 16, 47, 54]	327/476 (68.7)	6.57	3	0.09	54	2.06	0.99-4.28	0.05
Non-LC-HCC		93/165 (56.4)							
Non-LC-HCC	[12, 16, 47, 54]	93/165 (56.4)	7.60	3	0.05	61	2.16	1.08-4.32	0.03
CHB/ASC		225/466 (48.3)							

Abbreviations: HCC, hepatocellular carcinoma; LC, liver cirrhosis; CHB, chronic hepatitis B; ASC, asymptomatic HBsAg carriers; LC-HCC, cirrhotic hepatocellular carcinoma; Non-LC-HCC, non-cirrhotic hepatocellular carcinoma.

In addition, we performed a subgroup analysis to compare A1762T/G1764A dual mutations between ASC, CHB, LC and HCC patients. Compared to LC patients, HCC patients had a higher risk of A1762T/G1764A dual mutations (OR = 1.55, 95% CI = 1.06-2.26, *P* = 0.02, Table 3 and Supplementary Figure S2). Moreover, a subgroup analysis of four studies [12, 16, 47, 54] with a random-effects model revealed no statistically significant difference between cirrhotic HCC (LC-HCC) and non-cirrhotic HCC (non-LC-HCC) patients in terms of the frequency of A1762T/G1764A dual mutations (OR = 2.06, 95% CI = 0.99-4.28, *P* = 0.05, Table 3 and Supplementary Figure S3). This result should be reevaluated in studies with larger samples sizes. We performed another comparison of BCP dual mutations between non-LC-HCC and non-LC patients, including CHB and ASC patients. Using a random-effects model, a meta-analysis of four studies [12, 16, 47, 54] showed that the non-LC-HCC patients had a significantly higher frequency of A1762T/G1764A dual mutations compared with the CHB and ASC patients (OR = 2.16, 95% CI = 1.08-4.32, *P* = 0.03, Table 3 and Supplementary Figure S4). Given the above results, we assumed that BCP A1762T/G1764A dual mutations might promote hepatocarcinogenesis even without progression to cirrhosis.

Additionally, A1762T/G1764A dual mutations contributed to a higher risk of HCC occurrence compared with CHB patients (OR = 2.87, 95% CI = 1.96-4.20, *P* < 0.00001, Table 3 and Supplementary Figure S5). A similar trend in A1762T/G1764A dual mutations was found in HCC and ASC patients. HCC patients had a significantly higher frequency of A1762T/G1764A dual mutations than the ASC patients (OR = 5.59, 95% CI = 3.17-9.83, *P* < 0.00001, Table 3 and Supplementary Figure S6). In summary, the frequency of A1762T/G1764A dual mutations increased in this manner: from ASC to chronic disease, and then to HCC (Supplementary Table S1).

### A1762T/G1764A mutations in different HBV genotypes

Heterogeneity was significant among the included studies [12, 20, 30, 31, 39, 47], which described A1762T/G1764A dual mutations from chronic HBV infection in HBV genotypes B and C. Chronic HBV-infected patients with genotype B had a significant lower incidence of A1762T/G1764A dual mutations compared with those with genotype C (OR = 0.30, 95% CI = 0.18-0.52, *P* < 0.0001, Figure 2.1); no heterogeneity was found when we compared BCP dual mutations between HBV genotypes B and C in HCC patients (*P* = 0.27, I^2^ = 24%). Similarly, HCC patients with HBV genotype B had lower levels of A1762T/G1764A dual mutations compared with those with genotype C (OR = 0.34, 95% CI = 0.13-0.94, *P* = 0.04, Figure 2.2). In HBV genotype C subjects, BCP A1762T/G1764A dual mutations contributed to significantly higher risk for HCC developing compared with non-mutation ones (OR = 3.47, 95% CI = 2.28-5.27, *P* < 0.00001, Figure 3.1). Heterogeneity was significant between studies comparing HCC occurrence in HBV genotype B (*P* < 0.00001, I^2^ = 93%), no difference was found (*P* = 0.34, Figure 3.2).

**Figure 2:**
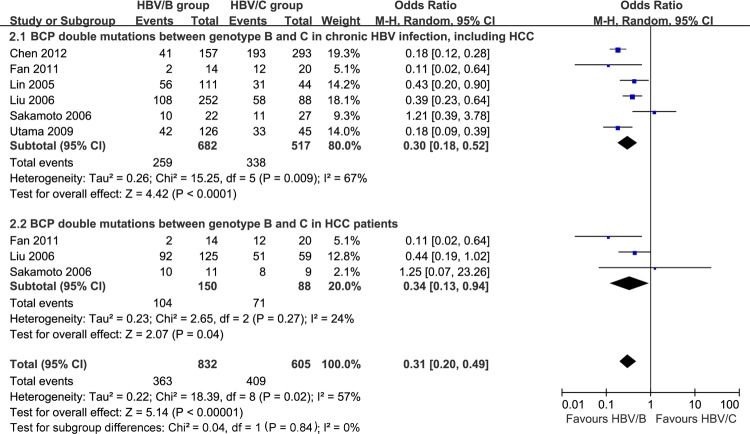
BCP A1762T/G1764A dual mutations in HBV genotype B and C from chronic HBV infection patients, including HCC

**Figure 3:**
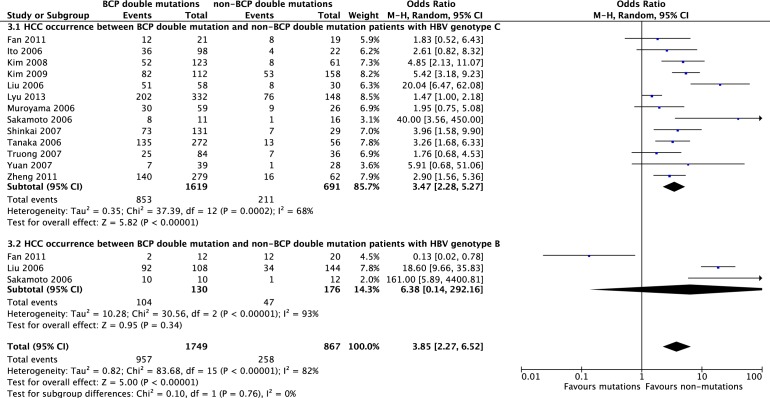
HCC occurrence between BCP A1762T/G1764A dual mutation and non-BCP dual mutation patients with HBV genotype C and genotype B

### A1762T/G1764A mutations from HBeAg-positive and HBeAg-negative patients

Nine studies [7, 12-14, 29, 37, 40, 42, 54] reported A1762T/G1764A dual mutations based on HBeAg status in chronic HBV infection. Heterogeneity was significant among these studies (P < 0.00001, I^2^ = 81%), and no significance in BCP A1762T/G1764A mutations was observed in either HBeAg-positive or HBeAg-negative chronic HBV-infected patients (OR = 1.06, 95% CI = 0.63-1.78, *P* = 0.83, Figure 4.1). In HCC, there was no difference of A1762T/G1764A mutations between HBeAg-positive and HBeAg-negative patients (OR = 1.38, 95% CI = 0.84-2.27, *P* = 0.20, Figure 4.2).

**Figure 4:**
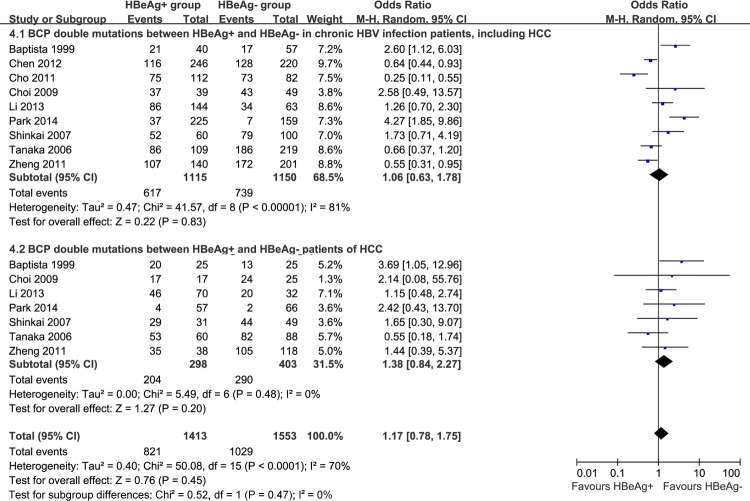
Comparison of BCP A1762T/G1764A dual mutations grouped by HBeAg status from chronic HBV infection patients, including HCC

## DISCUSSION

HCC is one of the most lethal cancers [56, 57]. The most common naturally occurring mutations in the BCP A1762T/G1764A dual mutation has been associated with hepatocarcinogenesis, but conflicts still exist [1,3]. Our meta-analysis showed that the A1762T/G1764A dual mutation was present at statistically significantly higher frequencies in HCC patients than in non-HCC controls, including LC, CHB and ASC patients. As the disease progressed, the risk of the A1762T/G1764A dual mutation in ASC, CHB, LC and HCC patients increased. Therefore, we hypothesized that A1762T/G1764A dual mutation might play a vital role in promoting disease progression in chronic HBV infection. Several previous case series have also shown a higher frequency of BCP T1762/A1764 mutation in patients with cirrhosis than in inactive carriers or patients with chronic hepatitis [16, 30, 58]. Our results confirmed this observation. However, the BCP A1762T/G1764A dual mutation is increasingly more prevalent as chronic HBV infection progresses from the asymptomatic HBsAg carrier state to liver cirrhosis or HCC, indicating that these mutations accumulate before the diagnosis of HCC. This finding suggests that BCP dual mutation may be a promising biomarker for predicting the clinical outcomes of patients with chronic HBV infection, particularly in terms of predicting whether they will develop HCC. Additionally, we performed a subgroup analysis that revealed no statistically significant difference in terms of the frequency of A1762T/G1764A dual mutations in liver cirrhotic HCC and non-cirrhotic HCC patients. Thus, we assumed that BCP A1762T/G1764A dual mutations might promote hepatocarcinogenesis even without progression to cirrhosis. Because the samples of non-cirrhotic patients with HCC in this investigation were relatively small, this hypothesis must be proven based on future studies with larger patient cohorts.

HBV genotype is associated with the risk of HCC. One possible reason for this association is that HBV mutations may be more common in some HBV genotypes than in others [2]. In this research, we found that chronic HBV-infected patients with genotype B had a significantly lower risk of A1762T/G1764A dual mutations compared with those with genotype C. In HCC, patients with HBV genotype C suffered higher A1762T/G1764A dual mutations compared with those with genotype B. Previous reports have shown that patients with genotype C infection have a higher positive rate of HBeAg, more severe liver disease and higher risk of developing HCC [30, 59-61]. On the basis of previous research and our results, we suggested that HBV genotype C patients tend to have a higher proportion of BCP T1762/A1764 mutations in conjunction with chronic HBV infection, including HCC [31, 58].

It has been suggested that the A1762T/G1764A double mutation in the BCP region can reduce the synthesis of HBeAg and enhance viral replication. However, this analysis found no significance of BCP A1762T/G1764A mutations from HBeAg-positive and HBeAg-negative chronic HBV-infected patients. The same finding was true for HCC patients. When introduced into wild-type HBV genomes, the BCP dual mutation indeed decreased HBeAg expression and enhanced viral genome replication of about twofold [60,62]. Transfection studies have shown that the T1762A/G1764A dual mutations decrease the level of pre-C mRNA by 50% to 70%, thereby inhibiting HBeAg production. A previous report showed that HBeAg titers are closely correlated with BCP mutation, and HBeAg-positive patients of genotype C infection had a higher prevalence of the A1762T/G1764A mutations [63]. Although no difference of BCP dual mutations was found in our meta-analysis, further research focusing on the mechanisms and the relationship between BCP mutation and HBeAg status is needed.

This meta-analysis had the following limitations. First, patients in the included studies had different HBV genotypes, different ethnicities, leading to significant heterogeneity; second, most of the included studies had small samples, with mid- to low-quality designs. In the future, high-quality, well-designed research focused on the mechanisms of BCP dual mutation in the progression of chronic HBV infection and HCC must be performed.

Based on our results, we could conclude that BCP A1762T/G1764A dual mutations are associated with disease progression and HCC occurrence in chronic HBV-infected patients. Additionally, this mutation might promote hepatocarcinogenesis even without progression to cirrhosis. Considered the frequency ratio of A1762T/G1764A dual mutation from ASC, CHB, LC and HCC, we suggested that BCP dual mutation should be screened in the CHB and LC patients.

## MATERIALS AND METHODS

### Search strategy and study selection

We searched PubMed, Ovid, Web of Science, and Cochrane Library databases for studies published to June 1, 2015. The following medical subject headings were used: “hepatocellular carcinoma;” “hepatitis B virus X protein;” “mutation;” “basal core promoter;” “A1762T;” “G1764A;” and “variation.” Electronic searches were supplemented with manual searches of reference lists used in all retrieved review articles, primary studies, and abstracts from meetings to identify other studies not found in the electronic searches. The literature was searched by two authors (Z Yang and L Zhuang) independently.

Two authors independently selected studies and discussed them with each other when inconsistencies were found. Articles that satisfied the following criteria were included: (1) case-control or cohort studies, (2) HCC and control subjects, including ASC, CHB or LC patients, (3) BCP A1762T/G1764A dual mutations (for HBV mutation), (4) HCC outcomes, and (5) available full texts. If the duration and sources of the study population recruitment overlapped by more than 30% in two or more papers by the same authors, we only included the most recent study or the study with the larger number of HCC patients. The following exclusion criteria were applied: (1) studies that included patients who were coinfected with hepatitis A, C, D, E virus or human immunodeficiency virus, had alcohol-related liver diseases, or had previously received antiviral treatments, (2) studies without control subjects, and (3) studies that only investigated A1762T or G1764A single mutations.

### Data extraction and methodological quality assessment

In our evaluation of the BCP A1762T/G1764A dual mutant, subjects with either a single mutation or a deletion at either site were not included in the analysis. Two researchers independently read the full texts and extracted the following information: publication data; study design; sample size; HBV genotypes; country or area; and study matching factors. The methodological qualities of the included studies were assessed according to the report by Liu et al. [64]. A 12-point scoring system was used that was based on factors that indicated good-quality observational studies, as shown in Table 1. Two authors (ZG Yang and LP Zhuang) independently assessed the study quality, and inconsistency was discussed with another reviewer-author (X Chen), who acted as an arbiter.

### Definitions

All diagnoses should be made according to the guidelines. ASC were defined as being seropositive for HBsAg for at least 6 months, having no evidence of LC or HCC based on the clinical criteria and undergoing ultrasound examination, with normal alanine aminotransferase (ALT). CHB was defined as being seropositive for HBsAg for at least 6 months, having persistent or intermittently elevated ALT levels or a liver biopsy showing chronic hepatitis with moderate or severe necroinflammation, with no clinical evidence of LC. The patients with LC were diagnosed by histologic analysis of liver biopsy specimens or by findings of repeated ultrasonography that were suggestive of cirrhosis and supplemented with clinical criteria indicating portal hypertension (i.e., the presence of ascites, thrombocytopenia, and esophageal varices). HCC was defined by at least one of the following criteria: (1) liver biopsy or (2) elevated alpha-fetoprotein levels and sonography, computed tomography or magnetic resonance imaging evidence.

### Statistical methods

The effect measures of interest were odds ratios (ORs) and the corresponding 95% confidence intervals (CIs). Heterogeneity across studies was informally assessed by visually inspecting forest plots and then formally estimated by Cochran's Q test in which chi-squared distribution was used to make inferences regarding the null hypothesis of homogeneity (considered significant at P < 0.10). A rough guide to our interpretation of I^2^ follows:

- 0% to 40% shows that heterogeneity may not be important
- 30% to 60% corresponds to moderate heterogeneity
- 50% to 90% indicates substantial heterogeneity
- 75% to 100% indicates considerable heterogeneity [65, 66]

If the eligibility of some studies in the meta-analysis was uncertain because of missing information, a sensitivity analysis was performed by conducting the meta-analysis twice: in the first meta-analysis, all studies were included; in the second meta-analysis, only studies that were definitely eligible were included. A fixed-effects model was initially used for our meta-analyses; a random-effects model was then used in the presence of heterogeneity. Description analysis was performed when the quantitative data could not be pooled. Review Manager software, version 5.1, was used for data analysis. All statistical tests were two-tailed, and differences with *P* < 0.05 were considered statistically significant.

## SUPPLEMENTARY MATERIAL FIGURES AND TABLE


