# Supplementary material for: Naturally occurring basal core promoter A1762T/G1764A dual mutations increase the risk of HBV-related hepatocellular carcinoma: a meta-analysis
Source: Oncotarget. 2016 Feb 2;7(11):12525–36. doi: 10.18632/oncotarget.7123 (PMC4914302; doi:10.18632/oncotarget.7123)
Supplement: Supplementary file 1 [file oncotarget-07-12525-s001.pdf]

Naturally occurring basal core promoter A1762T/G1764A dual mutations increase the risk of HBV-related hepatocellular carcinoma: a meta-analysis

Supplementary Material

Study or Subgroup	HCC group Events	HCC group Total	Non-HCC group Events	Non-HCC group Total	Weight	Odds Ratio M-H, Random, 95% CI
Asim 2010	25	39	6	29	1.8%	6.85 [2.25, 20.80]
Bahramali 2008	3	7	20	55	1.3%	1.31 [0.27, 6.47]
Bai 2011	111	152	91	136	2.5%	1.34 [0.81, 2.22]
Baptista 1999	33	50	5	47	1.8%	16.31 [5.45, 48.81]
Blackberg 2003	9	16	11	19	1.6%	0.94 [0.24, 3.58]
Chen 2006	38	50	52	102	2.2%	3.04 [1.43, 6.49]
Chen 2008	63	80	82	160	2.4%	3.53 [1.90, 6.55]
Chen 2009	16	40	5	20	1.7%	2.00 [0.61, 6.60]
Chen 2012	92	156	152	310	2.6%	1.49 [1.01, 2.20]
Cho 2011	63	69	85	125	2.0%	4.94 [1.97, 12.37]
Choi 2009	41	42	39	46	1.0%	7.36 [0.87, 62.59]
Chou 2008	39	71	19	81	2.3%	3.98 [1.99, 7.97]
Chu 2012	41	80	40	120	2.4%	2.10 [1.18, 3.75]
Constantinescu 2014	16	94	45	375	2.4%	1.50 [0.81, 2.80]
Datta 2014	17	22	23	46	1.8%	3.40 [1.07, 10.76]
Elkady 2008	3	23	3	25	1.2%	1.10 [0.20, 6.09]
Fan 2011	14	34	19	38	2.0%	0.70 [0.28, 1.78]
Guo 2008	38	58	52	71	2.2%	0.69 [0.33, 1.48]
Ito 2006	36	40	62	80	1.8%	2.61 [0.82, 8.32]
Jang 2007	5	6	12	23	0.9%	4.58 [0.46, 45.61]
Kao 2012	82	112	17	56	2.3%	6.27 [3.09, 12.71]
Kim 2008	52	60	71	124	2.1%	4.85 [2.13, 11.07]
Kim 2009	82	135	30	135	2.5%	5.42 [3.18, 9.23]
Kuang 2005	14	34	2	68	1.4%	23.10 [4.84, 110.34]
Lee 2011	27	31	47	65	1.7%	2.59 [0.79, 8.43]
Li 2013	77	102	58	105	2.4%	2.50 [1.38, 4.52]
Lin 2005	23	32	49	80	2.1%	1.62 [0.66, 3.95]
Liu 2006	153	200	23	160	2.4%	19.39 [11.19, 33.59]
Livingston 2007	6	47	6	32	1.7%	0.63 [0.18, 2.18]
Lyu 2013	202	278	132	204	2.6%	1.45 [0.98, 2.14]
Malik 2012	20	30	53	146	2.1%	3.51 [1.53, 8.05]
Muroyama 2006	30	39	29	36	1.8%	0.80 [0.26, 2.45]
Panigrahi 2012	15	20	11	69	1.7%	15.82 [4.76, 52.51]
Park 2014	6	132	38	310	2.1%	0.34 [0.14, 0.83]
Qu 2014	108	152	73	131	2.5%	1.95 [1.19, 3.19]
Shinkai 2007	73	80	58	80	2.0%	3.96 [1.58, 9.90]
Song 2005	4	48	0	105	0.6%	21.34 [1.12, 404.69]
Tanaka 2006	88	96	88	114	2.1%	3.25 [1.40, 7.57]
Tangkijvanich 2010	53	60	33	60	2.0%	6.19 [2.42, 15.83]
Tong 2007	73	101	10	67	2.2%	14.86 [6.67, 33.10]
Tong 2013	82	173	65	240	2.6%	2.43 [1.61, 3.66]
Truong 2007	25	32	63	88	2.0%	1.42 [0.54, 3.69]
Utama 2009	26	48	49	123	2.3%	1.78 [0.91, 3.50]
Wang 2007	40	47	62	164	2.1%	9.40 [3.97, 22.28]
Xu 2010	55	60	89	120	1.9%	3.83 [1.41, 10.44]
Yin 2011	152	190	360	1269	2.6%	10.10 [6.94, 14.71]
Yuan 2007	7	8	32	59	0.9%	5.91 [0.68, 51.06]
Yuan 2009	22	49	21	97	2.2%	2.95 [1.40, 6.19]
Zhang 2007	21	28	18	28	1.8%	1.67 [0.53, 5.28]
Zheng 2011	140	156	139	185	2.4%	2.90 [1.56, 5.36]
Zhu 2008	19	20	45	83	1.0%	16.04 [2.05, 125.47]
**Total (95% CI)**		**3729**		**6511**	**100.0%**	**3.05 [2.35, 3.95]**
Total events	2480		2594			

Heterogeneity: Tau² = 0.64; Chi² = 270.40, df = 50 (P < 0.00001); I² = 82%
Test for overall effect: Z = 8.42 (P < 0.00001)

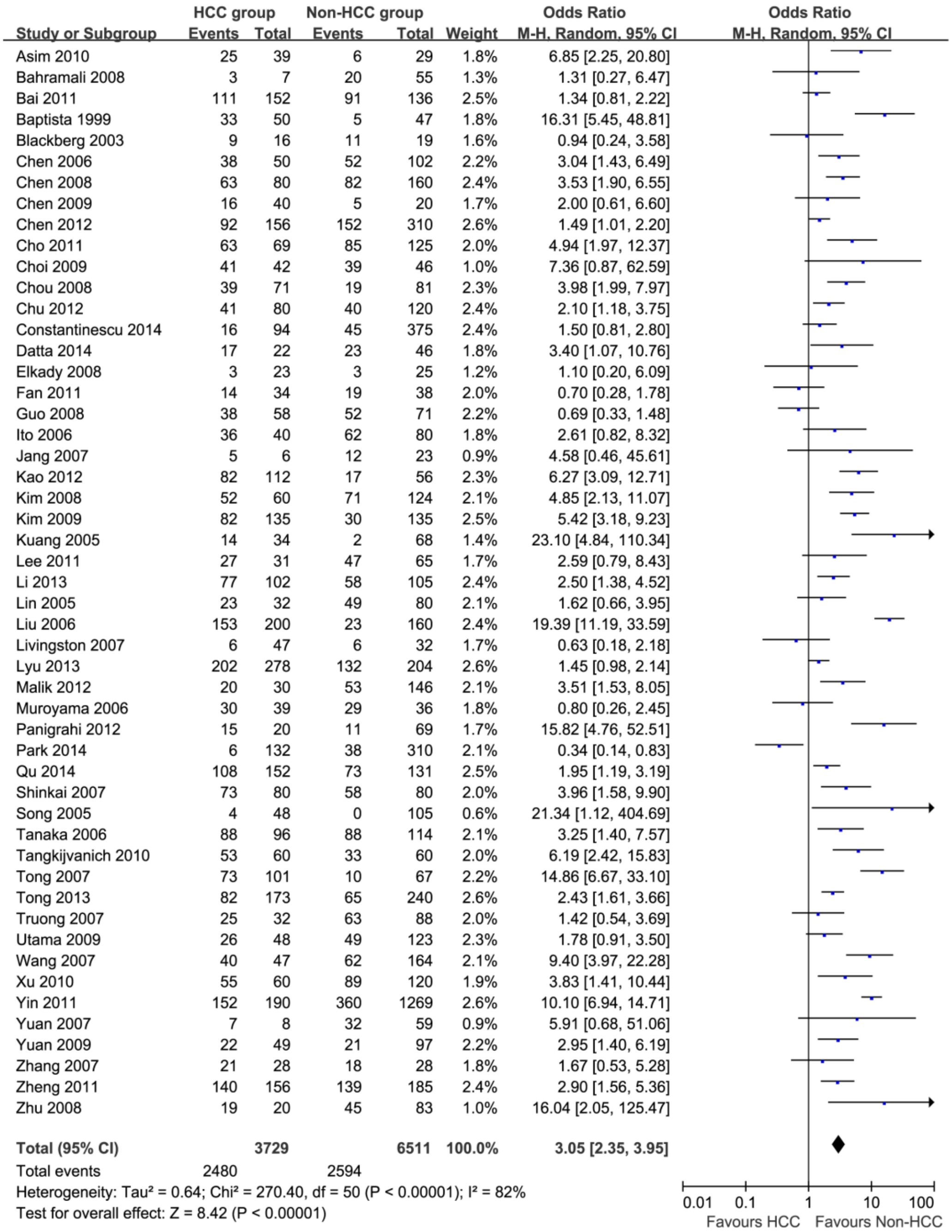

Figure S1 Frequency of BCP A1762T/G1764A dual mutations between HCC and non-HCC patients.

Study or Subgroup	HCC group Events	HCC group Total	LC group Events	LC group Total	Weight	Risk Difference M-H, Random, 95% CI
Bahramali 2008	3	7	4	13	1.8%	0.12 [-0.32, 0.57]
Chen 2006	38	50	17	18	6.5%	-0.18 [-0.34, -0.03]
Chen 2012	92	156	86	150	8.0%	0.02 [-0.09, 0.13]
Cho 2011	63	69	53	65	7.9%	0.10 [-0.02, 0.21]
Choi 2009	41	42	39	46	7.9%	0.13 [0.01, 0.24]
Chu 2012	41	80	32	60	6.2%	-0.02 [-0.19, 0.15]
Datta 2014	17	22	13	20	3.7%	0.12 [-0.15, 0.40]
Jang 2007	5	6	5	7	1.8%	0.12 [-0.33, 0.57]
Kim 2008	52	60	36	42	7.2%	0.01 [-0.13, 0.15]
Lee 2011	14	31	10	23	3.8%	0.02 [-0.25, 0.28]
Lin 2005	22	32	25	31	5.0%	-0.12 [-0.33, 0.09]
Malik 2012	20	30	14	30	4.2%	0.20 [-0.05, 0.45]
Panigrahi 2012	15	20	9	29	4.1%	0.44 [0.19, 0.69]
Song 2005	4	48	0	28	8.6%	0.08 [-0.01, 0.18]
Truong 2007	25	32	22	27	5.2%	-0.03 [-0.24, 0.17]
Utama 2009	26	48	37	62	5.7%	-0.06 [-0.24, 0.13]
Wang 2007	40	47	18	32	5.3%	0.29 [0.09, 0.49]
Yuan 2007	7	8	12	17	3.1%	0.17 [-0.15, 0.48]
Zhu 2008	19	20	9	18	4.1%	0.45 [0.20, 0.70]
**Total (95% CI)**		**808**		**718**	**100.0%**	**0.08 [0.01, 0.14]**
Total events	544		441			

Heterogeneity: Tau² = 0.01; Chi² = 43.21, df = 18 (P = 0.0007); I² = 58%

Test for overall effect: Z = 2.33 (P = 0.02)

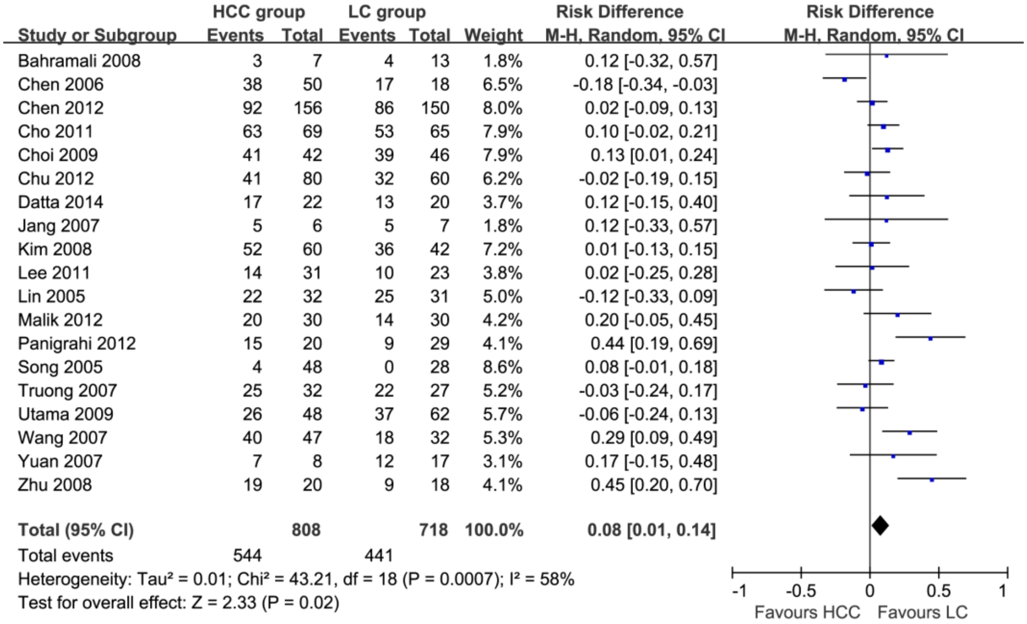

Figure S2 Frequency of BCP A1762T/G1764A dual mutations between HCC and LC patients.

Study or Subgroup	LC-HCC Events	LC-HCC Total	Non-LC-HCC Events	Non-LC-HCC Total	Weight	Odds Ratio M-H, Random, 95% CI
Chen 2012	61	90	31	66	34.0%	2.37 [1.23, 4.57]
Chu 2012	157	261	5	20	24.0%	4.53 [1.60, 12.84]
Utama 2009	5	12	21	36	18.4%	0.51 [0.14, 1.92]
Zheng 2011	104	113	36	43	23.6%	2.25 [0.78, 6.47]
**Total (95% CI)**		**476**		**165**	**100.0%**	**2.06 [0.99, 4.28]**
Total events	327		93			

Heterogeneity: Tau² = 0.30; Chi² = 6.57, df = 3 (P = 0.09); I² = 54%

Test for overall effect: Z = 1.94 (P = 0.05)

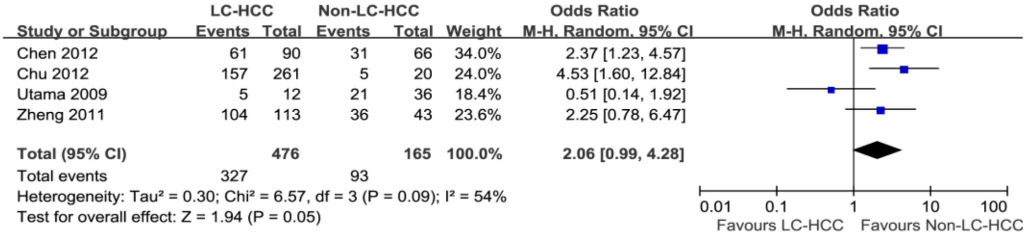

Figure S3 Frequency of BCP A1762T/G1764A dual mutations between LC-HCC and non-LC-HCC patients.

Study or Subgroup	Non-LC-HCC Events	Non-LC-HCC Total	CHB/ASC Events	CHB/ASC Total	Weight	Odds Ratio M-H, Random, 95% CI	Odds Ratio M-H, Random, 95% CI
Chen 2012	31	66	66	160	32.8%	1.26 [0.71, 2.25]	
Chu 2012	5	20	8	60	17.7%	2.17 [0.62, 7.61]	
Utama 2009	21	36	12	61	24.3%	5.72 [2.29, 14.28]	
Zheng 2011	36	43	139	185	25.2%	1.70 [0.71, 4.09]	
**Total (95% CI)**		**165**		**466**	**100.0%**	**2.16 [1.08, 4.32]**	
Total events	93		225				

Heterogeneity: Tau² = 0.29; Chi² = 7.60, df = 3 (P = 0.05); I² = 61%

Test for overall effect: Z = 2.19 (P = 0.03)

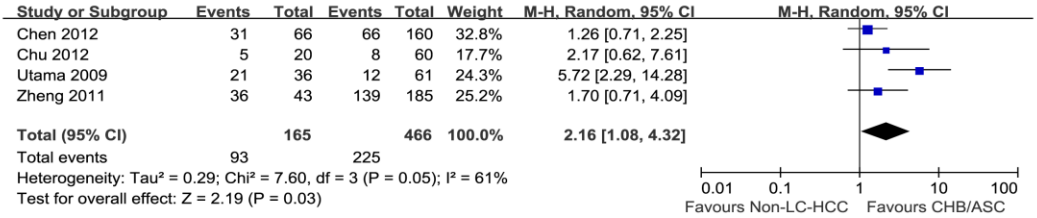

Figure S4 Frequency of BCP A1762T/G1764A dual mutations between non-LC-HCC and CHB/ASC patients.

Study or Subgroup	HCC group Events	HCC group Total	CHB group Events	CHB group Total	Weight	Odds Ratio M-H, Random, 95% CI
Bahramali 2008	3	7	11	30	3.1%	1.30 [0.24, 6.89]
Chen 2006	38	50	21	38	5.4%	2.56 [1.03, 6.38]
Chen 2012	92	156	66	160	7.1%	2.05 [1.31, 3.20]
Cho 2011	63	69	32	60	5.2%	9.19 [3.45, 24.46]
Fan 2011	14	34	19	38	5.3%	0.70 [0.28, 1.78]
Guo 2008	38	58	52	71	6.0%	0.69 [0.33, 1.48]
Ito 2006	36	40	31	40	4.2%	2.61 [0.73, 9.32]
Jang 2007	5	6	5	9	1.8%	4.00 [0.32, 49.60]
Kim 2008	52	60	25	41	5.2%	4.16 [1.57, 11.01]
Lee 2011	14	31	22	42	5.3%	0.75 [0.30, 1.90]
Li 2013	77	102	58	105	6.6%	2.50 [1.38, 4.52]
Lin 2005	22	32	24	49	5.3%	2.29 [0.90, 5.83]
Lyu 2013	202	278	132	204	7.3%	1.45 [0.98, 2.14]
Malik 2012	20	30	39	116	5.6%	3.95 [1.69, 9.25]
Panigrahi 2012	15	20	2	40	3.0%	57.00 [9.95, 326.49]
Song 2005	4	48	0	34	1.4%	6.98 [0.36, 134.03]
Utama 2009	26	48	12	61	5.6%	4.83 [2.06, 11.28]
Wang 2007	40	47	44	132	5.5%	11.43 [4.74, 27.57]
Yuan 2007	7	8	17	28	2.1%	4.53 [0.49, 42.05]
Zhang 2007	140	156	139	185	6.5%	2.90 [1.56, 5.36]
Zhu 2008	19	20	45	83	2.4%	16.04 [2.05, 125.47]
**Total (95% CI)**		**1300**		**1566**	**100.0%**	**2.87 [1.96, 4.20]**
Total events	927		796			

Heterogeneity: Tau² = 0.48; Chi² = 72.49, df = 20 (P < 0.00001); I² = 72%

Test for overall effect: Z = 5.42 (P < 0.00001)

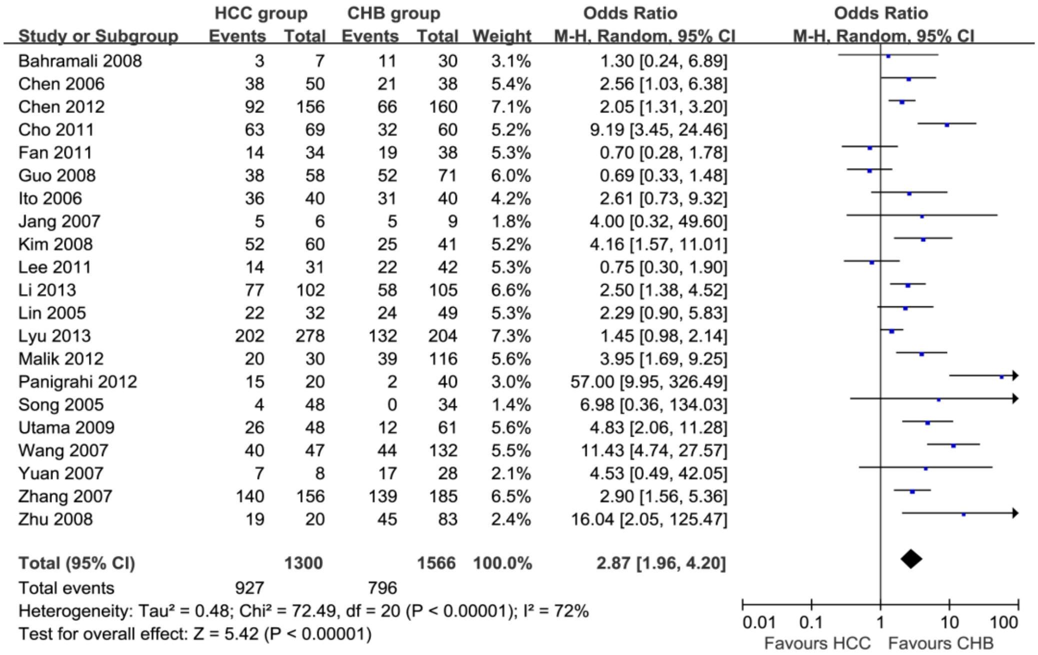

Figure S5 Frequency of BCP A1762T/G1764A dual mutations between HCC and CHB patients.

Study or Subgroup	HCC group Events	HCC group Total	ASC group Events	ASC group Total	Weight	Odds Ratio M-H, Random, 95% CI
Bahramali 2008	3	7	5	12	4.5%	1.05 [0.16, 6.92]
Baptista 1999	33	50	5	47	6.8%	16.31 [5.45, 48.81]
Blackberg 2003	11	16	9	19	5.9%	2.44 [0.61, 9.80]
Chen 2006	38	50	14	46	7.4%	7.24 [2.93, 17.86]
Chu 2012	41	80	8	60	7.5%	6.83 [2.88, 16.21]
Constantinescu 2014	16	94	45	375	8.2%	1.50 [0.81, 2.80]
Ito 2006	36	40	31	40	6.2%	2.61 [0.73, 9.32]
Jang 2007	5	6	2	7	3.0%	12.50 [0.84, 186.30]
Kao 2012	82	112	17	56	8.0%	6.27 [3.09, 12.71]
Kim 2008	52	60	10	41	7.0%	20.15 [7.19, 56.47]
Lin 2005	22	32	25	62	7.4%	3.26 [1.32, 8.04]
Liu 2006	153	200	23	160	8.4%	19.39 [11.19, 33.59]
Panigrahi 2012	15	20	39	50	6.4%	0.85 [0.25, 2.85]
Song 2005	4	48	0	12	2.6%	2.53 [0.13, 50.19]
Tong 2007	73	101	10	67	7.7%	14.86 [6.67, 33.10]
Yuan 2007	7	8	3	14	3.3%	25.67 [2.21, 298.49]
**Total (95% CI)**		**924**		**1068**	**100.0%**	**5.59 [3.17, 9.83]**
Total events	591		246			

Heterogeneity: Tau² = 0.92; Chi² = 70.33, df = 15 (P < 0.00001); I² = 79%

Test for overall effect: Z = 5.97 (P < 0.00001)

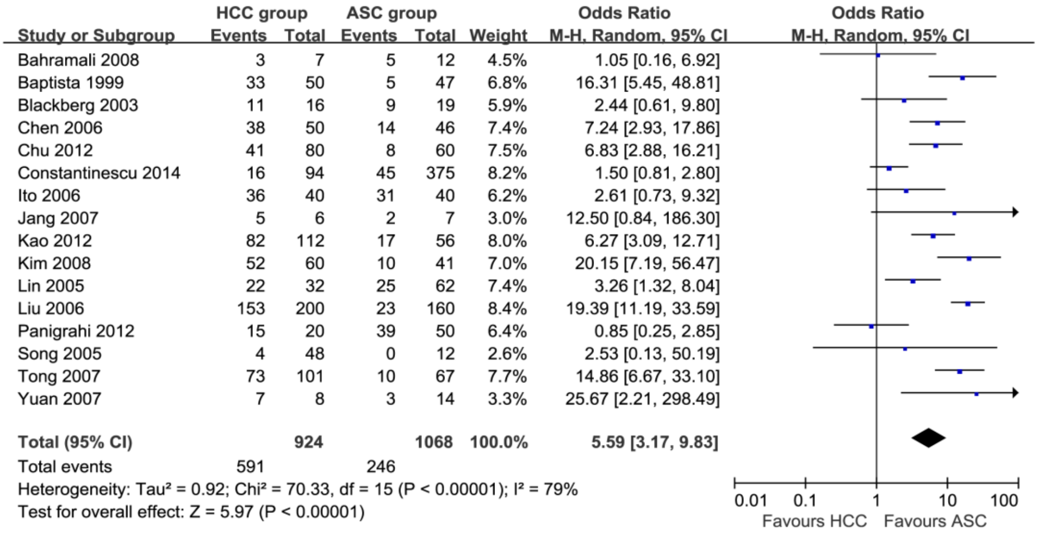

Figure S6 Frequency of BCP A1762T/G1764A dual mutations between HCC and ASC patients.

Table S1 Comparisons of A1762T/G1764A dual mutations from ASCs, CHB, LC and HCC.

Studies	Ref.	ASCs	CHB	LC	HCC
Bahramali 2008	[5]	5/12	11/30	4/13	3/7
Chen 2006	[9]	14/46	21/38	17/18	38/50
Jang 2007	[23]	2/7	5/9	5/7	5/6
Kim 2008	[25]	10/41	25/41	36/42	52/60
Lin 2005	[30]	25/62	24/49	25/31	22/32
Panigrahi 2012	[36]	39/87	2/8	9/22	15/19
Song 2005	[41]	0/12	0/34	0/28	4/48
Yuan 2007	[51]	3/14	17/28	12/17	7/8
Total		98/281 (34.9)	105/237 (44.3)	108/178 (60.7)	146/230 (63.5)
